# p12 Tethers the Murine Leukemia Virus Pre-integration Complex to Mitotic Chromosomes

**DOI:** 10.1371/journal.ppat.1003103

**Published:** 2012-12-27

**Authors:** Efrat Elis, Marcelo Ehrlich, Adi Prizan-Ravid, Nihay Laham-Karam, Eran Bacharach

**Affiliations:** Department of Cell Research and Immunology, The George S. Wise Faculty of Life Sciences, Tel Aviv University, Tel Aviv, Israel; Vanderbilt University School of Medicine, United States of America

## Abstract

The p12 protein of the murine leukemia virus (MLV) is a constituent of the pre-integration complex (PIC) but its function in this complex remains unknown. We developed an imaging system to monitor MLV PIC trafficking in live cells. This allowed the visualization of PIC docking to mitotic chromosomes and its release upon exit from mitosis. Docking occurred concomitantly with nuclear envelope breakdown and was impaired for PICs of viruses with lethal p12 mutations. Insertion of a heterologous chromatin binding module into p12 of one of these mutants restored PICs attachment to the chromosomes and partially rescued virus replication. Capsid dissociated from wild type PICs in mitotic cells but remained associated with PICs harboring tethering-negative p12 mutants. Altogether, these results explain, in part, MLV restriction to dividing cells and reveal a role for p12 as a factor that tethers MLV PIC to mitotic chromosomes.

## Author Summary

Retroviruses, including the murine leukemia virus (MLV), reverse transcribe their RNA genome to a DNA copy, which travels from the cytoplasm to the nucleus as part of a ‘pre-integration complex’ (PIC), to integrate into cellular chromosomes. The viral p12 protein is a constituent of the MLV PIC, but its function in this complex has remained unknown. We developed a real-time imaging system to detect p12 and MLV PICs in live cells. This revealed that p12 tethers the MLV PIC to mitotic chromosomes. Accordingly, PICs derived from viruses with specific lethal mutations in p12 failed to attach to the chromosomes, and insertion of a heterologous chromatin binding module into p12 restored PICs attachment to the chromosomes and rescued virus replication. In addition, docking of wild type PICs to chromosomes coincided with nuclear envelope breakdown during mitosis, and detachment occurred upon exit from mitosis. Capsid - another viral component of the PIC - dissociated from wild type PICs in mitotic cells but remained associated with PICs harboring tethering-negative p12 mutants, suggesting interplay between these two proteins in regulating targeting of mitotic chromosomes by the PIC. These results highlight steps contributing to the high tropism of MLV to dividing cells.

## Introduction

To integrate, reverse transcribed retroviral genomes are imported from the cytoplasm to the chromosomes as part of a pre-integration complex (PIC). Differences in retrovirus PIC trafficking influence their ability to infect resting and/or dividing cells. Lentiviruses, including the human immunodeficiency virus (HIV), infect both dividing and resting cells. In contrast, simple oncoretroviruses, such as murine leukemia viruses (MLV), are restricted to dividing cells [1], [2], [3], [4]. The HIV PIC is capable of entering the nucleus through the nuclear pore complexes, allowing integration in chromosomes of resting cells. The MLV PIC is thought to get access to the chromosomes only during mitosis, upon nuclear envelope (NE) disassembly, as inferred from correlating kinetics of cell division and integration [3].

This dissimilarity between HIV and MLV PIC trafficking likely stems from their different composition [4]. Capsid (CA), present in MLV PICs [5], [6], [7] and absent from HIV PICs [8], [9], contributes to the difference in ability to transduce nondividing cells [10]. Also, the lens epithelium-derived growth factor (LEDGF/p75), interacts with HIV PICs [11], [12] tethers the integrase (IN) of HIV and other lentiviruses to chromatin [11], [13], [14], [15], [16], but not the MLV IN [11], [17], for which no equivalent tethering factor has been identified.

p12, a cleavage product of MLV Gag precursor, is thought to influence MLV integration. p12 acts in the budding of assembled Gags, and mutations in this domain hamper particle morphogenesis and release [18], [19], [20]. Viruses with other lethal mutations in p12 showed early infection defects, with normal generation of linear genomic DNA, but no circular DNA forms [18], [19], [21], [22]. The latter forms, thought to be generated by nuclear enzymes, are not substrates for IN-mediated integration but mark nuclear entry of the viral DNA. Their absence in cells infected with some p12 mutants, and the normal *in vitro* integration activity shown by the PIC of one of these mutants [18], suggest that p12 functions in an unknown way after reverse transcription and before integration.

p12 is a component of the MLV PIC, crucial for the progression of the PIC towards integration: p12, observed as discrete puncta, associates with CA and the viral genomic DNA and this p12- genome association occurs in the cytoplasm and adjoining chromosomes, suggesting that p12 escorts the viral genomic DNA throughout early stages of infection [7]. p12-containing PICs accumulate on mitotic chromosomes, however this accumulation is impaired for p12 proteins with a mutation that rendered the virus integration-defective [7]. These data implied that p12 functions in directing the PIC to integration, yet its precise role has remained unknown. Here we imaged MLV-PICs in live cells and revealed that p12 tethers the MLV PIC to mitotic chromosomes.

## Results

### Establishing an imaging system to detect MLV p12/PICs in live cells

**Figure 1:**
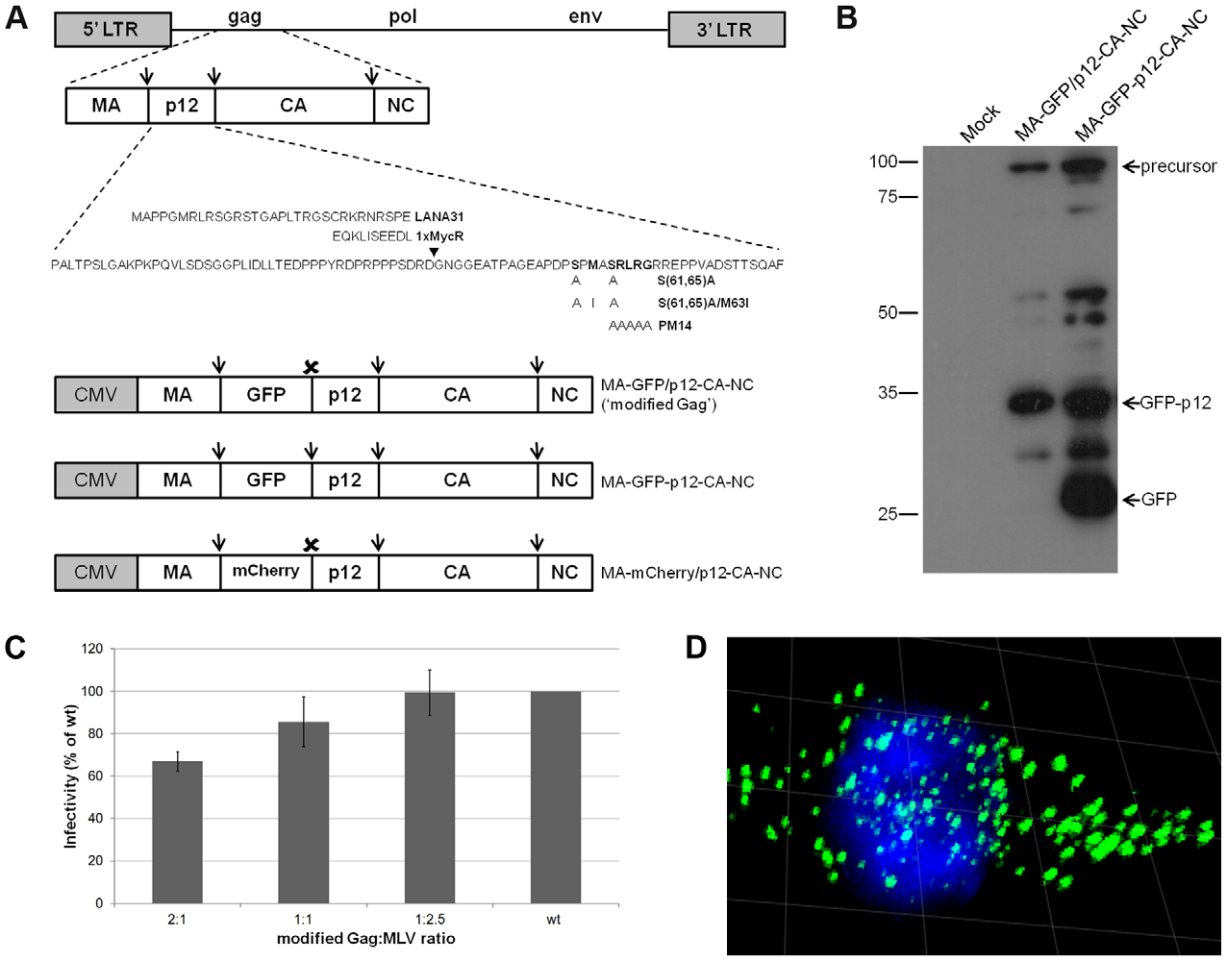
Characteristics of real time imaging system for MLV PICs. (A) Schematics of MLV genome, Gag, modified Gags, p12 and mutations. Mutated residues are aligned with the cognate wt residues (in bold). Arrowhead marks LANA31 and Myc epitope insertion site. Arrows and ✗ represent protease-cleavage sites and protease-resistant linker (GGSI), respectively. (B) Western blot of chimeric virions. Anti-GFP antibody was used to detect the processing of GFP fusion proteins in virion pellets, purified from supernatants of cultures, transfected with the indicated modified Gags and wt MLV. Mock represents transfection with no plasmid DNA. (C) Infectivity of chimeric virions. The indicated molar ratios of MA-GFP/p12-CA-NC and wt MLV plasmids were co-transfected into 293T cells, together with pQCXIP-GFP-C1 vector. Virions in culture supernatants, normalized by RT activity, were used to infect NIH3T3 cells, which were analyzed by FACS for GFP fluorescence, 2 days post-infection. Average infectivity from 3 independent experiments is presented as percentage of infectivity of wt particles with no modified Gag (wt). Error bars indicate SEM. (D) Confocal fluorescence microscopy of wt GFP-infected U/R cell. Serial optical sections were reconstituted into a 3D image, with a 10 µm grid. PICs are in green and Hoechst-stained chromosomes in blue.

To investigate the role of p12 in PIC trafficking we labeled the PIC with p12 fused to enhanced green fluorescent protein (GFP). An in-frame insertion of GFP to the central region of p12 was lethal to the virus (data not shown). Thus, we generated chimeric virions, composed of both wt and modified Gag molecules; the latter containing GFP fused to p12 N-terminus. In the modified Gag (MA-GFP/p12-CA-NC), the GFP sequence was inserted in-frame, downstream of the protease-cleavable matrix (MA)-p12 junction, and upstream of a short, non-cleavable linker fused to p12 (Fig. 1A). MA-GFP/p12-CA-NC retains wt p12-CA and CA-nucleocapsid (NC) cleavage sites, critical for MLV particle formation, maturation and infectivity [23], [24]. Wt virus was co-expressed with MA-GFP/p12-CA-NC, and virions were purified by ultracentrifugation through a 25% sucrose cushion and immunoblotted with anti-GFP antibody. This revealed a major band corresponding to the GFP-p12 fusion (∼36 kDa), a fainter band of MA-GFP/p12-CA-NC precursor (∼88 kDa) and traces of additional cleavage products (Fig. 1B); suggesting that MA-GFP/p12-CA-NC co-assembled with wt Gag and was processed by the viral protease. Importantly, the linker connecting GFP and p12 was protease-resistant as no free GFP was processed from MA-GFP/p12-CA-NC, unlike the control construct (named MA-GFP-p12-CA-NC, Fig. 1B), in which the GFP was flanked by cleavable sites (Fig. 1A).

To test if co-expression of MA-GFP/p12-CA-NC with wt virus affects infectivity, a MLV vector (pQCXIP-gfp-C1), encoding for the puromycin-resistance gene and GFP was expressed together with different ratios of MA-GFP/p12-CA-NC to wt virus. Virions, normalized by reverse transcriptase (RT) activity, were used to infect NIH3T3 cells and GFP^+^ cells were counted by fluorescence-activate cell sorting (FACS). A 1∶1 molar ratio resulted in only a minor reduction (∼15%) in infectivity compared to the wt virus (with no MA-GFP/p12-CA-NC) control (Fig. 1C). Counting the number of puromycin-resistant colonies in infected cultures gave similar results (data not shown). Thus, 1∶1 molar ratio was used in further experiments.

At 12 hr post-infection (hpi) of U/R cells (human osteosarcoma U2OS cells, expressing the murine receptor for MLV; [7]) with labeled chimeric virions (hereinafter named wt GFP), discrete fluorescent puncta were detected in the cytoplasm of interphase cells, and adjacent to the condensed chromosomes of mitotic cells (Fig. 1D). This appearance was identical to former images of PICs, labeled with Myc-tagged p12 proteins (derived from the replication-competent 1xMycR clone) [7]; in addition, 65±3% of the fluorescent puncta (approximately 100 dots/cell in 5 cells were analyzed) overlapped the chromosomes in mitotic cells, in good agreement with the 70% overlap between Myc-tagged p12 proteins and mitotic chromosomes, as was quantified before [7]. Immunostaining of wt GFP-infected U/R cells with antibodies against CA (a component of the MLV PIC) revealed extensive co-localization between GFP and CA signals (Fig. S1A); and quantification of the overlap between these two signals revealed a 71±4% overlap in interphase cells (data was obtained from five cells, each containing ∼80 fluorescent dots). This number is similar to the extent of overlap measured for Myc-tagged p12 and CA in interphase U/R cells, infected with 1xMycR virus (∼80%; see below). This suggests that GFP-p12 molecules are associated with the PICs to a similar extent as p12 molecules lacking the GFP moiety. Thus, the GFP-p12 labeling system successfully marks the incoming PICs.

### MLV PICs show both undirected and directed movements in the cytoplasm of interphase cells and are unable to cross the NE

To monitor cytoplasmic-nuclear trafficking of PICs in live cells, we labeled the NE of U/R cells by stably expressing lamin A fused to red fluorescent protein (RFP-lamin A; U/R/RFP-laminA cells) [25]. U/R/RFP-laminA cells, arrested before S phase by serum starvation and aphidicolin treatment [3], were infected with wt GFP virions. At 18 hpi, GFP-labeled PICs exhibited undirected and directed movements, including towards the NE, in sharp contrast to the immobility of virions attached to the cover-slip (Movie S1). None of the PICs crossed the intact NE (Movie S1 and see below), demonstrating the physical barrier that the intact NE imposes on nuclear entry of the PICs in interphase cells - a notion previously deduced from measuring integration kinetics, in respect to the cell cycle [3], but never directly shown.

### MLV PICs dock to mitotic chromosomes

**Figure 2:**
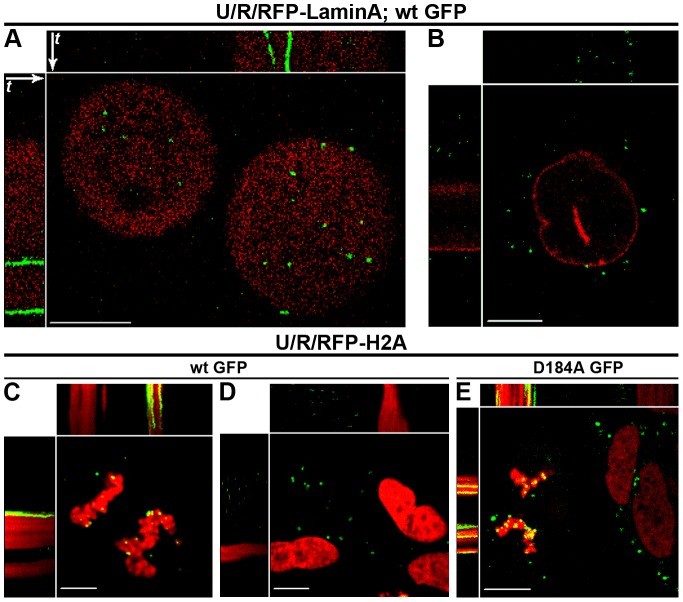
MLV PICs dock to mitotic chromosomes independently of IN activity. U/R/RFP-laminA (A, B) and U/R/RFP-H2A (C–E) cells were infected with wt GFP (A–D), or with D184A GFP (E) virions. Shown are representing kymographs (A–E) of cells imaged in Movie S2. White arrows (shown only in A but apply to all kymographs of all figures) represent the order of the movie frames over time (*t*); where *t* = 30, 47, 152, 150 and 26 seconds for A, B, C, D and E, respectively. In kymographs, immobile PICs appear as continues green lines (A, C, E); in contrast, motile PICs change x, y coordinates over time, resulting in the scattered/dotted pattern (B, D). Bars represent 10 µm.

To monitor the PICs in mitotic cells, U/R/RFP-laminA cells were arrested at metaphase with 2-methoxyestradiol (2ME2) and infected with wt GFP virions. 2ME2 impairs microtubule dynamics without gross microtubule depolymerization and arrests cells at the spindle assembly checkpoint [26], [27], [28]. Arrested cells displayed diffuse RFP-lamin A (indicating NE disassembly [29]) and restricted PIC motility (Fig. 2A; Movie S2, part A). In 2ME2-treated cells that did not reach metaphase (with intact NEs), PICs were restricted to the cytoplasm and motile (Fig. 2B; Movie S2, part B), excluding a direct 2ME2-induced inhibition of PIC motility.

To test for the docking of immobile PICs to mitotic chromosomes, we generated U/R/RFP-H2A cells [U/R cells stably expressing RFP fused to histone H2A (RFP-H2A)], infected them with wt GFP virions and imaged unsynchronized, interphase and mitotic cells. Indeed, motionless PICs were attached to the mitotic chromosomes, while cytoplasmic PICs in interphase cells were motile (Fig. 2C, D; Movie S2, parts C, D). Attachment of PICs to mitotic chromosomes was also observed for wt GFP-infected, unsynchronized mouse NIH3T3 cells expressing RFP-H2A (NIH3T3/RFP-H2A; Movie S2, part E); and for wt GFP-infected U/R/RFP-H2A cells that were arrested at M phase by nocodazole treatment (Movie S2, part F). In the latter settings, PICs attachment to mitotic chromosomes could also be observed in cells arrested at mitosis for up to 40 hpi (data not shown; extended time points were not tested because of apparent drug-induced cytotoxicity).

### Docking to mitotic chromosomes is independent of IN activity

The apparent docking of the PIC to the chromosomes may be the result of a stable association of the PIC components with the viral DNA genome that had integrated into the chromosomes. To test this, we made chimeric virions (named D184A GFP), using MLV with the D184A mutation in the catalytic site of IN, which disrupts its activity [30]. D184A GFP PICs were motile in interphase U/R/RFP-H2A cells, and docked to mitotic chromosomes in dividing cells (Fig. 2E; Movie S2, part G). Thus, IN activity is not required for the docking of MLV PICs to the chromosomes.

### PICs' docking to mitotic chromosomes coincides with NE breakdown

**Figure 3:**
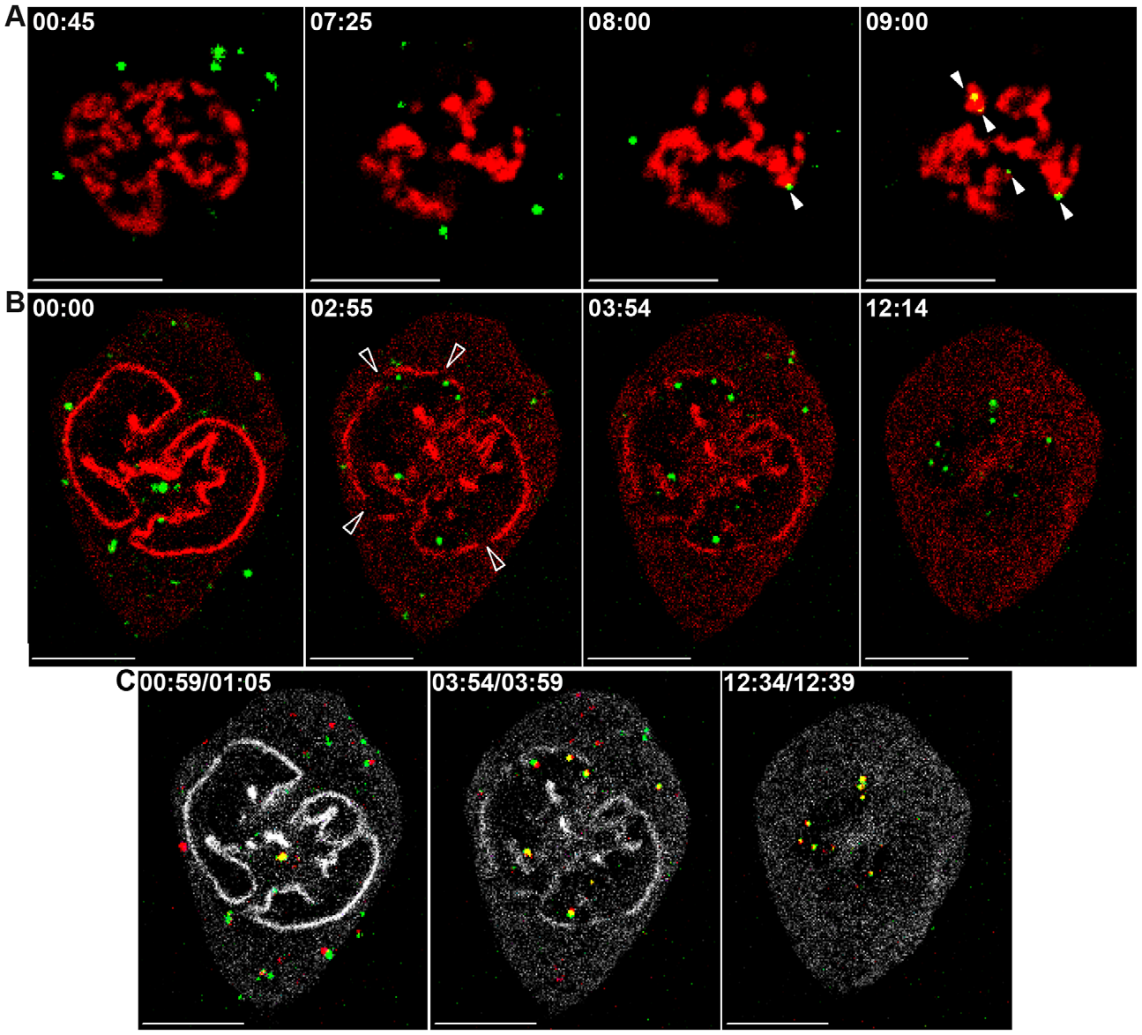
Docking onto mitotic chromosomes coincides with NE breakdown. wt GFP-infected U/R/RFP-H2A (A) and U/R/RFP-laminA (B, C) cells, were imaged upon entry to mitosis. Shown are frames from the resulting movies (Movie S3; parts A and B). Full arrowheads (A) mark PICs (green) anchored to the mitotic chromosomes (red). Empty arrowheads (B) mark gaps in the NE (red). (C) Visualization of PICs' movement during NE breakdown. Three pairs of frames were chosen from the start, middle and end of Movie S3, part B. For each pair, the PICs in the second frame were superimposed on the first frame and pseudo-colored with red. Lamin A signal was pseudo-colored with white. PICs with a relative large shift in their position appear in red or green while PICs with a minimal shift appear in yellow. Time (minutes and seconds) from start of imaging is shown for each frame. Bars represent 10 µm.

To monitor the transition between the cytoplasmic movements of the PICs to their docking to mitotic chromosomes, we viewed infected cells as they entered mitosis. Confluent U/R/RFP-H2A and U/R/RFP-laminA cells were infected with wt GFP particles and 2 hr later, the cells were trypsinized and replated at a lower density. After additional 5 hr we detected infected cells that entered mitosis as judged by the growing condensation of their chromosomes (U/R/RFP-H2A), or by the progressive dissolution of the NE (U/R/RFP-laminA). Docking of the GFP-labeled PICs to the RFP-labeled chromosomes could readily be detected; the time frame between the first observed docking event and the docking of the rest of the PICs was ∼2 min (Fig. 3A; Movie S3, part A). Similarly, the immobilization of PICs occurred concomitantly with the breakdown of the RFP-labeled NE (Fig. 3B, C; Movie S3, part B), in contrast to the cytoplasmic PICs that remained mobile in the same cells (Fig. 3C; Movie S3, part B). Thus, these images are consistent with the idea that the intact NE might act as a physical barrier to the PICs.

### MLV PICs dock to mitotic but not interphase chromosomes

**Figure 4:**
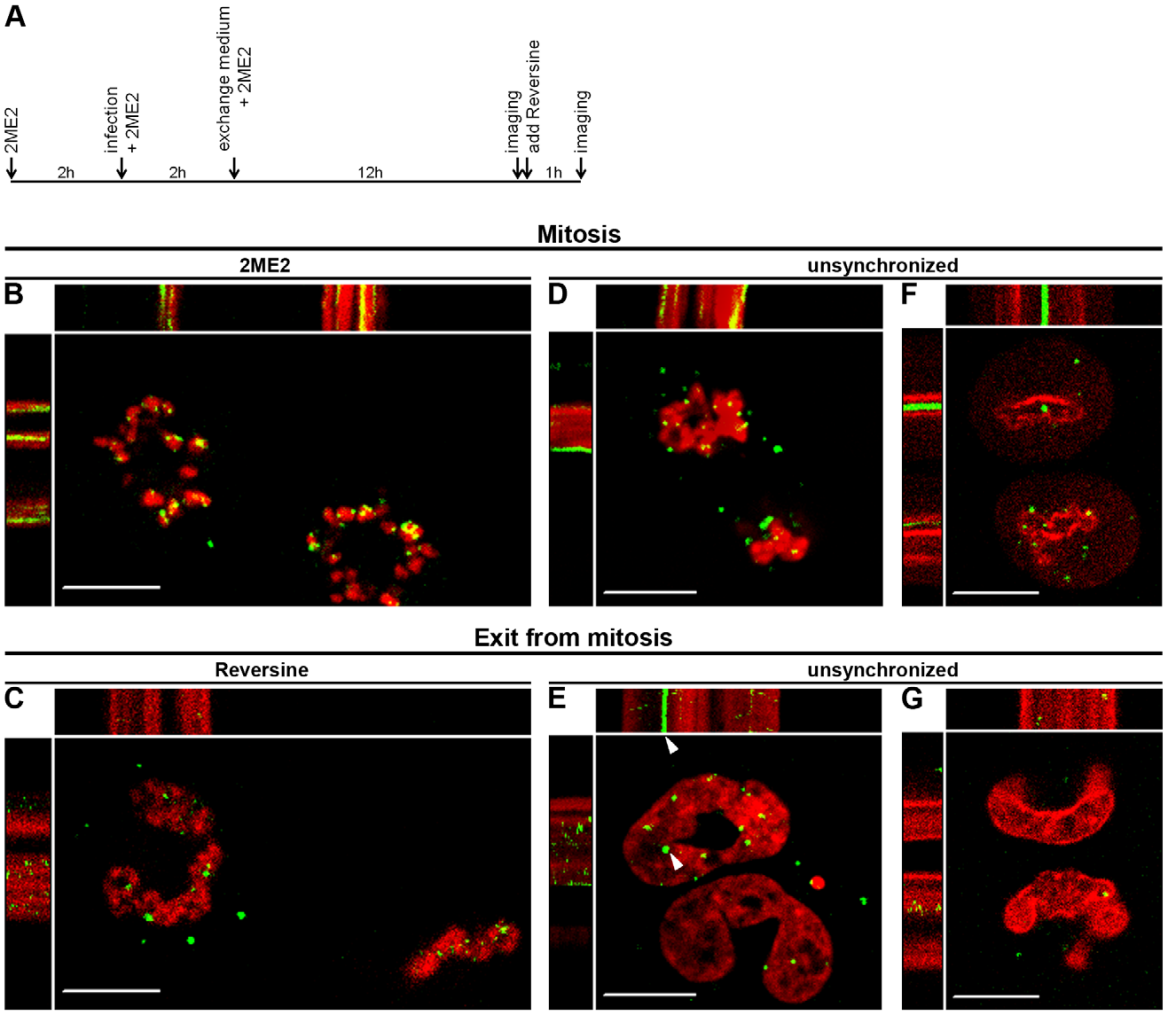
Release of PICs from chromosomes upon exit from mitosis. (A) Timeline of 2ME2 and Reversine treatments. Reversine was added immediately after completion of the first imaging session. (B–G) Representing kymographs of wt GFP-infected cells (Movie S4), imaged for 16 (B, C) or ∼30 (D–G) seconds. The same cells were imaged at mitosis and exit from mitosis. (B, C) U/R/RFP-H2A cells, treated as in (A). (D, E) Infected, unsynchronized U/R/RFP-H2A, or (F, G) U/R/RFP-laminA, cells at mitosis (D, F) and exit of mitosis (E, G). The cells in E and G were imaged ∼4 and 1 hr, respectively, after their initial imaging. Arrowheads mark a PIC that remained associated with the chromosome. Bars represent 10 µm.

To probe if PICs bind exclusively to mitotic chromosomes, provided that the physical barrier of the NE is avoided, we infected 2ME2-arrested U/R/RFP-H2A cells with wt GFP virions and imaged chromosome-docked PICs at 12 hpi. Addition of Reversine, which inhibits the Mps1 kinase, counters the spindle assembly checkpoint [31], and reverses the 2ME2-induced blockage of the cell cycle [32], resulted in the decondensation of the mitotic chromosomes within 1 hr (Fig. 4A–C; Movie S4, parts A, B). Remarkably, in these conditions, PICs regained their movement, which was now restricted to the nucleus (compare Fig. 4B to C, and part A to B in Movie S4). The same was also observed for unsynchronized U/R/RFP-H2A or U/R/RFP-laminA cells that naturally exit mitosis (without 2ME2/Reversine treatment; Fig. 4D–G; Movie S4, parts C–F).

The regaining of PICs movement during the exit from mitosis may reflect their release from the chromatin upon completion of the integration step. However, the same dissociation occurred also with D184A GFP PICs that are unable to discharge their viral genomic DNA due to the lack of IN activity (compare the same D184A GFP-infected U/R/RFP-H2A cells in Movie S2 part G and Movie S4 part G). This result provides further support for the affinity of the MLV PIC towards mitotic, and not interphase, chromosomes. Of note, a minor fraction of PICs (integration-competent wt GFP or integration-incompetent D184A GFP) remained associated with the chromatin following exit from mitosis, (Movie S4; and see an example in Fig. 4E, marked with an arrowhead); demonstrating that integration is not a pre-requisite for such a stable association.

### Real-time imaging of p12 mutant PICs reveals a role for p12 in tethering the PICs to the chromatin

**Figure 5:**
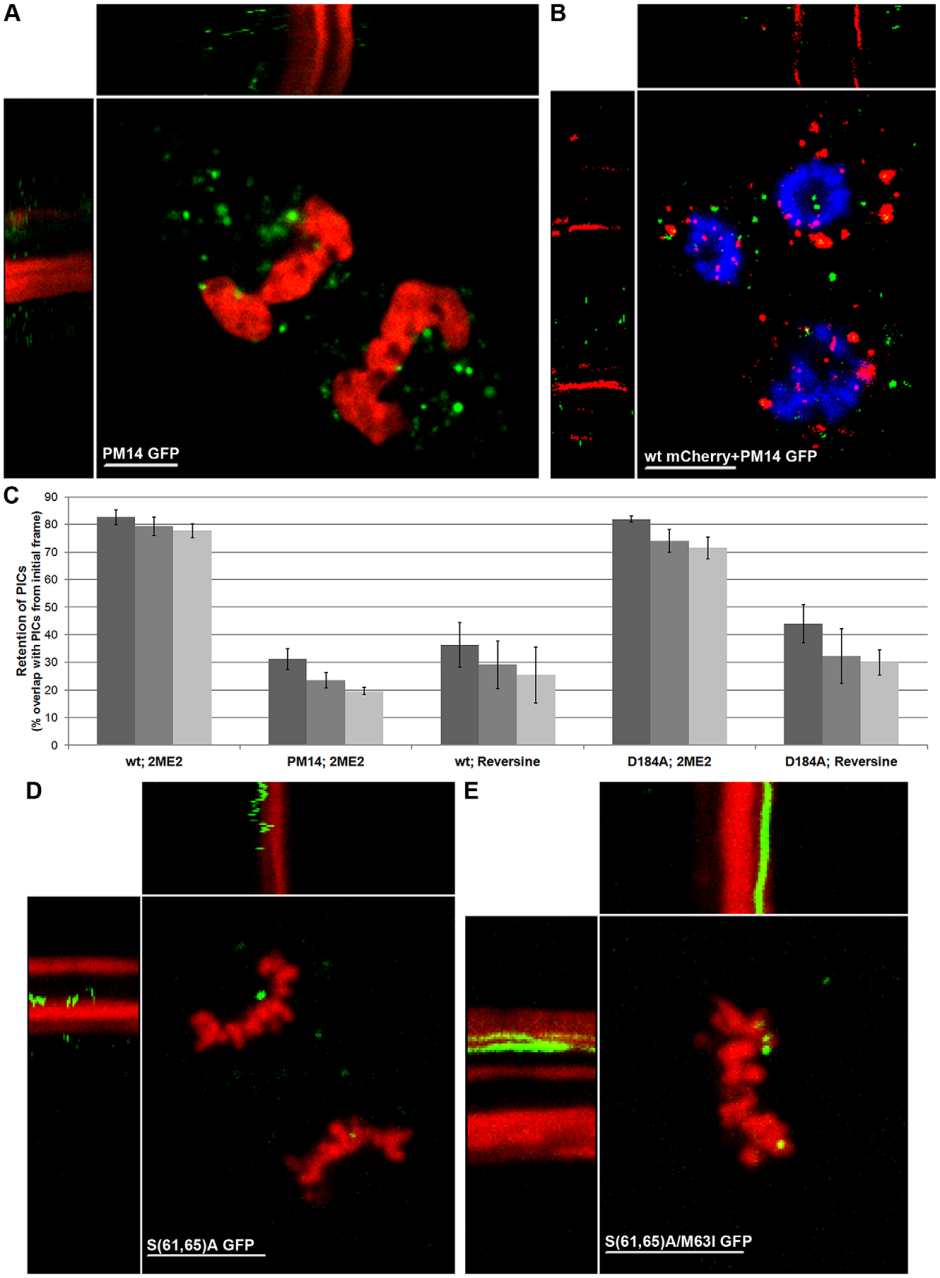
Failure of p12 mutants to dock to mitotic chromosomes. (A, B, D and E) Representing kymographs of unsynchronized, mitotic U/R/RFP-H2A (A, C and D) or 2ME2-arrested U/R (B) cells infected with PM14 GFP (A), PM14 GFP and wt mCherry (B), S(61,65)A GFP (D), S(61,65)A/M63I GFP (E) shown in Movie S5. Hoechst-stained chromosomes are in blue (B). *t* = 134, 74, 48 and 48 seconds for A, B, D and E, respectively. Bars represent 10 µm. (C) Quantification of spatial retention of PICs over time. The percentages of overlap between PICs in the first frame and PICs in the second, third and fourth frames (dark, intermediate and lighter bars, respectively) was calculated for wt GFP (wt), PM14 GFP (PM14) and D184A GFP (D184A) -infected cells, treated with the indicated drug. All the PICs in the above frames were identified through intensity-based segmentation (Materials and Methods), and analyzed with no selection for specific PICs. Shown are the average values obtained from 6 (wt; 2ME2), 8 (PM14; 2ME2), 4 (wt; Reversine), 7 (D184A; 2ME2) and 4 (D184A; Reversine) inspected cells, each with approximately 30 PICs, taken from 4, 4, 3, 4 and 2 movies, respectively. For all treatments, the time between frames ranged from 0.6 to 0.9 seconds. Error bars indicate SEM. Bars represent 10 µm.

The above imaging employed p12 as a marker of PICs, but falls short of attributing a function to p12. Defined mutations in p12 [such as a five-amino acids alanine block substitution, named PM14; and the S61A and S(61, 65)A mutations] are lethal to the virus and dramatically reduce the levels of circular forms of the viral genomic DNA, suggesting a defect in nuclear entry of this genome [18], [19], [22]. Myc-labeled PM14 PICs are normally distributed in the cytoplasm of interphase cells, but fail to accumulate on mitotic chromosomes [7]. We next generated GFP-labeled mutant chimeric virions (PM14 GFP; composed of PM14 virus and modified Gags with PM14 mutation) and infected and imaged (at 12 hpi) unsynchronized U/R/RFP-H2A cells. In the cytoplasm of interphase cells, PM14 GFP PICs moved similarly to wt GFP PICs (Movie S5, part A). A portion of PM14 PICs reached the chromosomes in mitotic cells; however, despite their proximity to chromosomes, all were motile and none attached to the mitotic chromosomes (Fig. 5A; Movie S5, part B). The failure in docking was also observed in unsynchronized, mitotic mouse NIH3T3/RFP-H2A cells (Movie S5, part C). This strongly implies a role for p12 in the docking of MLV PICs to the chromosomes. To monitor wt and PM14 PICs in the same cell we generated chimeric virions (named wt mCherry), labeled with mCherry instead of GFP (using the MA-mCherry/p12-CA-NC construct; Fig. 1A). In Hoechst-stained 2ME2-arrested U/R cells, PM14 GFP PICs were motile, in contrast to the immobilization of the wt mCherry PICs on condensed chromosomes (Fig. 5B; Movie S5, part D). To quantify the spatial retention of PICs over time we calculated the percentage of overlap between the PICs in the first frame, to the PICs in the second, third and fourth frames of each movie (Fig. 5C). While high retention (∼70 to 80%) was observed for wt GFP and D184A GFP PICs over time; PM14 GFP PICs showed lower and decreasing retention overtime (∼30 to 20%). This low retention was comparable to that of motile wt GFP and D184A GFP PICs upon Reversine-mediated exit from mitosis (Fig. 5C). For PM14 GFP, we also quantified the overlap between the signals of GFP and mitotic chromosomes in fixed cells, and found it to be approximately 16±2% (approximately 100 dots/cell in 8 cells were analyzed). This value is in a good agreement with both the relative low retention exhibited by this mutant in the above real-time analysis, and the low overlap (11%) measured before for Myc-tagged p12 proteins and mitotic chromosomes [7]. This relatively low overlap is in contrast to the 63% overlap, measured for wt GFP and mitotic chromosomes in fixed cells (see above). Reconstitution of serial optical sections into 3D images of mitotic chromosomes of U/R/RFP-H2A cells further showed the close contacts between such chromosomes and wt GFP (Fig. S2A), but not PM14 GFP (Fig. S2B), PICs.

Of note, chimeric PICs resulting from the expression of wt MLV genome and modified Gag containing the PM14 mutation docked to mitotic chromosomes (not shown), demonstrating that the docking activity of wt p12 is dominant over the lack of such activity of PM14 GFP-p12, and providing genetic evidence for the ability of GFP-p12 to mark the incoming MLV PIC.

Viruses carrying the S(61,65)A mutations are phenotypically undistinguishable from the PM14 virus. However, a replication-competent revertant with an additional compensatory mutation (M63I) in p12 exists for this virus [22]. We introduced the S(61,65)A or the S(61,65)A/M63I to the 1xMycR clone and to the modified Gag (Fig. 1A) and co-expressed each cognate pair of constructs to generate chimeric virions [named S(61,65)A GFP or S(61,65)A/M63I GFP, respectively]. S(61,65)A GFP PICs failed to stably anchor to mitotic chromosomes or showed a very short, unstable association with the chromosomes (Fig. 5D; Movie S5, part E). The S(61,65)A/M63I PICs, in contrast, stably docked to mitotic chromosomes, identically to wt PICs (Fig. 5E; Movie S5, part F). A 25 and 65% overlap with mitotic chromosomes was measured for S(61,65)A GFP and S(61,65)A/M63I GFP, respectively; in good accord with the 10 and 65% overlap of PM14 and wt viruses, respectively [7]. Altogether, these results further demonstrate the correlation between the replication competence of the tested virus and the ability of its PIC to dock to the chromosomes; and provide additional demonstration for the connection between the impairment of such docking and the presence of specific mutations in p12.

### Insertion of a heterologous chromatin-binding module into mutant p12 restores PIC docking and partially rescues viral infectivity

**Figure 6:**
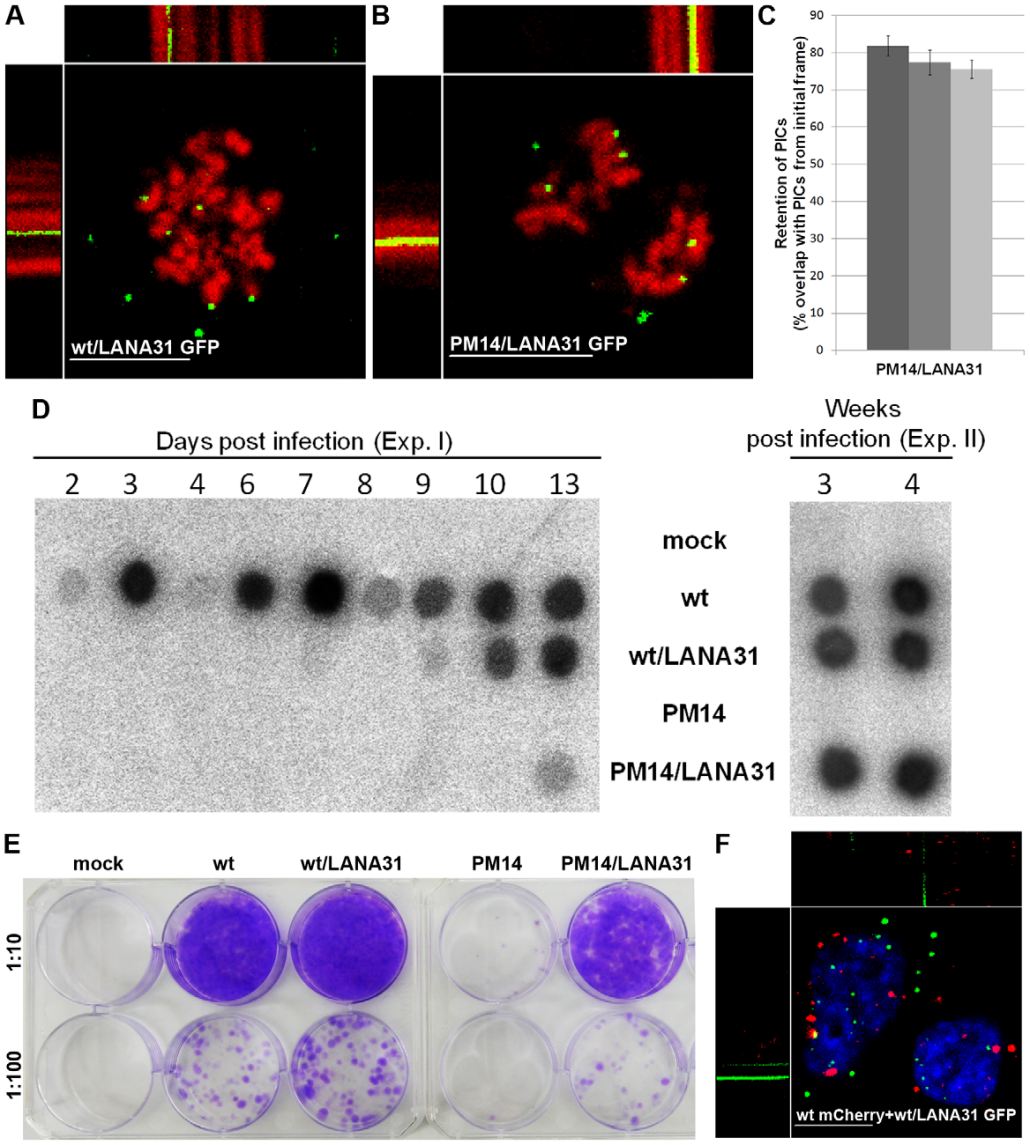
LANA31 insertion into p12 of PM14 virus rescues PIC docking to chromosomes and viral replication. Representing kymographs from Movie S6 of unsynchronized, mitotic U/R/RFP-H2A (A, B) or U/R cells that exit mitosis (F), infected with wt/LANA31 GFP (A), PM14/LANA31 GFP (B), or wt mCherry together with wt/LANA31 GFP (F). Hoechst-stained chromosomes are in blue (F). *t* = 24, 24 and 72 seconds for A, B and F, respectively. Bars represent 10 µm. (C) Quantification of spatial retention of PICs over time. Retention of PM14/LANA31 GFP was calculated as described for Fig. 5C. Shown are the average values obtained from four cells, each from an independent movie, and each with approximately 20 PICs. Error bars indicate SEM. (D) Virus spreading. NIH3T3 cells were infected with the indicated viruses, normalized by RT activity. Samples of culture supernatants were harvested at the indicated time points and assayed for RT activity to detect virus spreading. Mock represents uninfected cells. Shown are results of two experiments (Exp. I and II). (E) Single-cycle infection assay. VLPs, harboring the indicated modifications, and normalized by exogenous RT assay, were used to transduce the pQCXIN vector into NIH3T3 cells. 2 dpi the cells were diluted (1∶10 and 1∶100) and selected in G418 medium for additional 10 days. G418-resistant colonies were fixed and stained with crystal violet. Shown is one of three independent experiments.

To evaluate if addition of a foreign chromatin-binding element rescues the docking of mutant p12 PICs, we inserted such a module of the herpes LANA protein (LANA31), into p12 of the PM14 clone, generating the PM14/LANA31 virus. LANA31 consists of 31 residues, 23 of which bind the groove between histones 2A and 2B [33]. This module restores the tethering activity of a mutated LEDGF/p75, resulting in the binding of HIV IN to chromatin [34]. A control virus (named wt/LANA31) was also made by inserting LANA31 into p12 of wt MLV. LANA31 was inserted between the DRD and GNG residues of p12 (Fig. 1A), as MLV replication tolerates insertion of a Myc tag into this location [7]. These viruses were expressed with the MA-GFP/p12-CA-NC modified Gag, resulting in chimeric viruses (named wt/LANA31 GFP and PM14/LANA31 GFP). PICs of PM14/LANA31 GFP stably anchored to mitotic chromosomes in U/R/RFP-H2A cells (Fig. 6B; Movie S6, part A) similarly to wt/LANA31 GFP (Fig. 6A; Movie S6, part B) and wt GFP (Fig. 2C; Movie S2, part C) PICs. Quantification of the overlap between the signals of GFP and mitotic chromosomes in fixed cells, revealed a 73±5% value for PM14/LANA31 GFP (approximately 70 dots/cell in 7 cells were analyzed), which was similar to the overlap found for wt GFP (63%), and which was in contrast to the low overlap (16%) measured for PM14 GFP (see above). In addition, quantification of the spatial retention of PM14/LANA31 GFP PICs over time in mitotic cells showed high retention levels that were almost identical to the retention of wt GFP PICs in mitotic, 2ME2-treated cells (compare Fig. 6C to 5C). Thus, insertion of the LANA31 peptide into p12 rescues chromatin docking of the PICs with PM14 mutations. To evaluate the effect of LANA31 insertion into p12 on virus infectivity we infected NIH3T3 cell cultures and monitored the kinetics of virus spread. Whereas wt virus spread quickly, wt/LANA31 showed much slower spreading (Fig. 6D), indicating that the insertion of LANA31 greatly attenuated virus replication. This is in line with our previous observation, showing that insertion of a peptide with a similar size (30 residues of a triple Myc epitope) in the same location in p12 attenuates virus replication [7]. Furthermore, sequence analysis of p12 of the viruses that spread in the wt/LANA31-infected cells revealed that LANA31 was rapidly deleted from wt/LANA31 virus (data not shown), substantiating the deleterious effect of this sequence on the virus. PM14 virus showed no spreading (here and [19]); the PM14/LANA31 virus showed detectable, slow spreading (Fig. 6D), indicating that insertion of LANA31 into p12 partially restored the infectivity of PM14 mutant. Sequence analysis of p12 of viruses that spread in the PM14/LANA31-infected cells showed a mixture of PM14/LANA31 sequence together with wt sequences (no PM14 and no LANA31; data not shown). This likely reflects the selection that the PM14 mutation enforces on the retention of the LANA31 sequence in p12 and the parallel recombination of the PM14/LANA31 slow virus with endogenous retroviruses of the mouse genome [35] that harbor wt p12 sequences. Such recombination, which likely involves the co-packaging of the endogenous and exogenous viral genomes, and the recombination between these genomes during reverse transcription [36], cannot efficiently occur with PM14 virus that lacks detectable replication, but may occur upon the multiple cycles of infection of the PM14/LANA31 virus.

Further support for the rescue of PM14 infectivity by LANA31 came from single-cycle infection assays, in which NIH3T3 cells were infected with wt, wt/LANA31, PM14 or PM14/LANA31 virus-like particles (VLPs), harboring puromycin or neomycin -resistance markers, and normalized by RT activity (Fig. 6E). Quantification of the number of drug-resistant cell colonies from three independent experiments revealed that LANA31 insertion into wt p12 reduced particles' infectivity to 85±11% of that of wt particles. A major increase in VLPs infectivity, however, was observed when LANA31 was inserted into PM14 p12: whereas PM14 VLPs had only residual infectivity (0.3±0.4%, compared to wt), PM14/LANA31 VLPs showed higher infectivity (16±8%, compared to wt). These results are in accordance with the spreading assays described above. Altogether, insertion of LANA31 into p12 compensates for PM14 effect on both virus replication and anchorage of the PIC to the chromosomes, further emphasizing the role of p12 as the tethering factor for the MLV PIC.

We also tested how the exit from mitosis affects LANA31-mediated docking of the PICs to the chromosomes. In unsynchronized U/R cells co-infected with wt/LANA31 GFP and wt mCherry, and stained with Hoechst; wt mCherry PICs regained their movement upon exit from mitosis, while wt/LANA31 GFP PICs remained motionless (Fig. 6F; Movie S6, part C). This result provides further support for the affinity of the wt PICs towards mitotic but not interphase chromatin.

Altogether, the above results demonstrate a role for p12 as a factor that tethers the MLV PIC to mitotic chromosomes.

### CA-p12 dissociation occurs in mitotic cells for wt, but not for p12 mutant viruses

**Figure 7:**
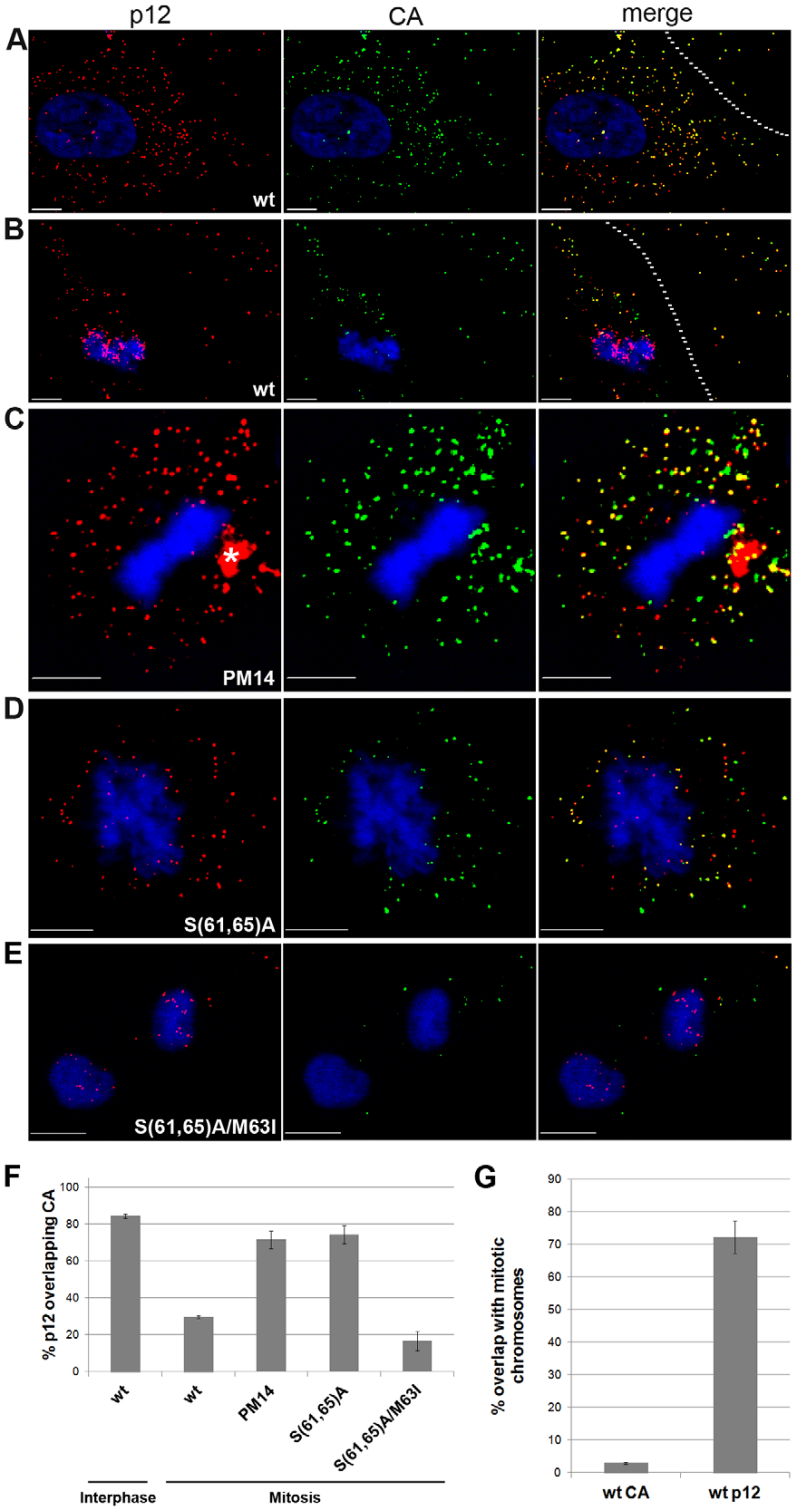
CA-p12 dissociation in mitotic cells: discrepancy between wt and p12 mutants. Immunofluorescence of interphase (A) and mitotic (B–E) U/R cells, infected with 1xMycR (A, B), or with 1xMycR clones carrying PM14 (C), S(61,65)A (D) and S(61,65)A/M63I (E) mutations. 12 hpi cells were stained with anti-CA (green) and anti-Myc (red) antibodies. Chromosomes were stained with DAPI (blue). Extracellular virions are to the right of the dashed lines (A, B). An asterisk (C) marks non-typical p12 staining. (F) Quantification of the percentage of p12 signal that overlaps CA signal in interphase and mitotic cells (∼400 p12 dots/cell were analyzed in 4 and 3 1xMycR-infected mitotic and interphase cells, respectively; ∼40–130 dots/cell in 5 cells were analyzed for the rest of the viruses). (G) Quantification of the percentage of p12 and CA signals that overlap the DAPI-stained chromatin in 1xMycR-infected mitotic cells. Error bars indicate standard error of the mean (SEM). Bars represent 10 µm.

CA and Myc-tagged p12 co-localize in the cytoplasm of interphase cells [7]. To further study the spatial relations between CA and p12, we compared interphase with mitotic 1xMycR-infected U/R cells, using immunofluorescence (Fig. 7). In interphase cells, the majority of the PICs were cytoplasmic with a clear overlap (∼80%) between CA and p12 signals, similar to the co-localization observed in particles attached to the glass outside the cells (Fig. 7A and see identical results in [7]). In contrast, in mitotic cells, p12 associated with the condensed mitotic chromosomes and almost no CA could be co-detected in these chromosome-localized p12 spots (∼30% overlap between p12 and CA, Fig. 7B, F; ∼70 and ∼3% overlap with the mitotic chromosomes for p12 and CA, respectively, Fig. 7G). Similar results were obtained when the overlap between CA and GFP-labeled PICs was measured (32±5%, calculated from three inspected cells, each containing ∼80 dots; Fig. S1B). These results, together with the fact that p12 co-localizes with the genomic viral DNA both in cytoplasmic PICs and in PICs attached to mitotic chromosomes [7], imply that during early stages of infection gradual uncoating events occur, involving the sequential dissociation of MA and CA, and the trafficking of p12 proteins as part of the PIC, to the chromosomes in mitotic cells. Importantly, in tethering-negative PM14 and S(61,65)A mutant PICs, a continued CA-p12 association was observed in mitotic cells (Fig. 7 C, D, F), while the S(61,65)A/M63I revertant showed wt levels of dissociation (Fig. 7E, F). This shows that lack of CA dissociation correlates with inability of p12 mutants to tether to mitotic chromosomes.

## Discussion

To follow MLV PICs by live cell microscopy, we generated chimeric particles co-assembled from wt Gag molecules and Gag harboring a viral-protease resistant GFP-p12 fusion. This labeling method was applied as p12 escorts the viral genomic DNA as part of the PIC to the chromosomes [7]; and since the chimeric particles showed only a slight reduction in infectivity. The notion that GFP-p12 labeled the PICs is supported by: (i) the similarity between punctuate distributions of GFP-p12 and Myc-p12 [7] in infected cells; (ii) the cytoplasmic localization of GFP-p12 puncta in interphase cells and their accumulation on mitotic chromosomes, identical to the patterns observed with fluorescence in situ hybridization (FISH)-labeled MLV genomes [3], [7]; (iii) the lack of chromosomal localization of MA-GFP/p12-CA-NC Gag proteins when exclusively assembled as VLPs (without wt Gag) and pseudotyped with the MLV ecotropic envelope (data not shown); (iv) the accumulation on mitotic chromosomes of GFP-p12 harboring the PM14 mutation, only when employed in the context of chimeric PICs containing wt p12 proteins; (v) the extent of overlap between CA and GFP-p12 signals, which was very similar to the overlap of CA and Myc-tagged p12 proteins (derived from the 1xMycR replication-competent virus), in both interphase and mitotic cells. Thus, our live-cell imaging system allows monitoring of early stages of MLV infection, and complements the envelope-based system, developed to detect MLV-cell interactions before entry [37]; and the systems developed to detect HIV PICs [38], [39].

Our main finding is the inability to dock to mitotic chromosomes of PICs derived from PM14 and S(61,65)A p12 mutants, which sharply contrasted to the stable docking of PICs from wt and the S(61,65)A/M63I revertant viruses. This was best visualized upon the co-infection of PM14 and wt viruses, which showed lack of docking, or stable chromosomal association, respectively, in the same cell. Moreover, insertion of a heterologous chromatin-binding module (LANA31) into p12 of the PM14 virus rescued both its infectivity and the anchorage of its PIC to the chromosomes, strongly implying a chromosome-tethering function for p12. The discrepancy between the full restoration of docking to the chromosomes of PM14/LANA31 PICs and the partial restoration of infectivity of this virus likely results from the adverse effect caused by the addition of 31 residues into p12 on other stages of the replication cycle; for example, we observed a reduction in assembly of the wt/LANA31virus (data not shown). A similar-sized insertion (a triple Myc epitope) in the same location also attenuated replication [7]. Of note, while the PM14 mutation in p12 resulted in complete disruption of PIC docking to the mitotic chromosomes, S(61,65)A PICs showed short, unstable associations with the chromosomes; possibly explaining why revertants could be isolated for the latter mutant [22]. Importantly, although the function of p12 as a chromatin tether can be deduced from the clear differences in the ability of the PICs described above to attach to the chromosomes, our microscopic analysis cannot distinguish between functional and nonfunctional PICs in a given cell.

LEDGF/p75 was identified as the factor that tethers the HIV IN to chromatin: it interacts with unknown chromatin ligands and with IN, and is essential for the chromosomal targeting of HIV IN [11], [14], [40]. LEDGF/p75 depletion hampers HIV integration and blocks infection of HIV and other lentiviruses; however, LEDGF/p75 does not interact with MLV IN and accordingly, its depletion does not affect MLV infection [11], [17], [41], [42], [43]. We suggest that different lentiviruses, including HIV, evolved to tether their PICs to the chromosomes through IN-LEDGF/p75 interactions; in contrast, MLV evolved to tether its PIC via a LEDGF/p75-independent way, which involves the use of p12. The notion of different tethering mechanisms of HIV and MLV is further supported by their differences in integration site selection [42], [43], [44], [45], [46], by similarity in the target site selection of a HIV chimeric virus expressing the MLV IN with MLV, and by the increased similarity upon replacement of the HIV Gag of this chimera with MLV Gag [47], [48]. Our data point to p12 as a Gag protein essential for MLV integration; but fall short of dissecting a putative role for p12 in target selection. A likely scenario is that p12 tethering activity is an essential prerequisite for integration, while progression to complete integration depends on additional interactions of IN with cellular factors [49].

What kind of interactions may be involved in the p12-mediated tethering activity? The specific recognition of mitotic chromosomes by p12-labled PICs, and their detachment upon exit from mitosis suggest that the MLV PIC recognizes chromatin features, such as post-translational modifications that are associated with cell cycle progression. For example, phosphorylation of histone H3 at Thr3 (H3T3) and methylation of the adjacent Lys4 (H3K4), serve as a motif for the binding of cellular factors, such as TFIID, to mitotic chromosomes [50]. Intriguingly, strong association between the target site selection of MLV and specific chromatin modifications, including H3K4 methylation, has been described [48]. However, no chromatin binding modules that recognize these modifications [51] have been identified in p12. Notably, p12 displays similarity to histone H5 protein [52] and may directly interact with chromatin. Alternatively, p12 may recruit a cellular factor harboring chromatin recognition domains.

MLV integration peaks in cells after exit from metaphase and decondensation of chromosomes [3]. The release of the GFP-p12 complexes from the decondensed chromosomes in cells that exit mitosis may represent the release of PICs after integration. However, the released GFP-p12 puncta may represent PICs that failed to integrate. The release of D184A PICs upon exit from mitosis exemplifies the independence of this release from IN activity. Moreover, the lack of release of LANA31-tethered PICs further underscores the specific affinity of wt MLV PICs for mitotic chromosomes. Of note, a minority of the wt PICs remained immobilized at the end of mitosis. Although it can be argued that only such PICs mediate active integration, this is unlikely as the same phenomenon was observed for D184A PICs.

Using immunofluorescence analysis, we demonstrated here that CA, a known component of the PIC [5], [6], co-localized with cytoplasmic p12 in interphase cells, but not with chromosome-docked p12 in mitosis. This result concords with the higher ratio between CA and viral genomic DNA, or CA and IN, in MLV PICs extracted from cytoplasmic fractions, compared to nuclear PICs -suggesting that CA is lost from the PIC after its entry into the nucleus [6]. Our results extend this finding and show that CA-PIC/p12 dissociations occur specifically upon mitosis, when no intact NE exists in the cell. Furthermore, the co-localization of p12 and the viral genomic DNA adjacent to the chromosomes [7] provides support for the notion that p12, in contrast to CA, associates with the PIC till the final stages of PIC trafficking. Thus, gradual uncoating events that depend on the cell cycle can be described for MLV: first, MA, which forms the protein layer adjacent to the internal side of the virion membrane, dissolves away after fusion of this membrane with the plasma membrane [7]. Next, CA, which forms an inner protein layer in the virion and is part of the PIC, dissociates from this complex in mitotic cells. In HIV, CA is also mainly absent from the PIC [8], [9]. Moreover, swaps between Gag domains of HIV and MLV demonstrated that CA is the dominant determinant for the difference between HIV and MLV in the ability to transduce nondividing cells and led to the suggestion that the stable association of the MLV CA with the PIC prevents the access of this complex to components of the cellular transport machinery [10]. Thus, the timely dissociation of CA from the PIC in mitotic cells, demonstrated here, may expose this complex to interactions with cellular factors, necessary for the completion of the trafficking and/or p12-mediated docking of the PIC to mitotic chromosomes. This notion is further supported by the correlations between the maintenance of CA association with the p12/PIC in PM14 and S(61,65)A mutants and their inability to dock to the chromosomes; and the reversal of both phenomena in the context of wt and S(61,65)A/M63I revertant. These data are in line with the proposed cooperative effect of p12 and CA at early stages of MLV infection [53].

In summary, we identified p12 - a PIC component essential for integration - as a factor that tethers the MLV PICs to mitotic chromosomes. MLV-based vectors have been used successfully in gene therapy trials in humans, yet with the risk of leukemogenesis [54], [55]. Identification of factors influencing integration, such as p12, should lead to safer MLV-derived vectors [49]. The specific docking of MLV PICs to mitotic chromosomes, the requirement for NE disassembly and the disassociation of CA from the PIC during mitosis, may all contribute to the productive integration of MLV in dividing cells.

## Materials and Methods

### Viruses and plasmids

Moloney MLV clones wt (pNCS), PM14, 1xMycR, S(61,65)A, S(61,65)A/M63I, and pQCXIP-gfp-C1 vector were described before [7], [19], [22]. LANA31 peptide (of the Kaposi's sarcoma herpesvirus LANA protein; obtained from R. Sarid, Bar-Ilan University), or mutations S(61,65)A and S(61,65)A/M63I, were introduced into the indicated viruses, as described before for the PM14 mutation [7]. Overlapping PCR was used to generate the sequences of MA-GFP/p12-CA-NC, MA-GFP-p12-CA-NC and MA-mCherry/p12-CA-NC (Supporting Information). pEF-H2AmRFP, expressing RFP-histone H2A fusion, and pRFP-lamin A, expressing RFP-Lamin A fusion [25], were provided by M. Brandeis (The Hebrew University of Jerusalem) and H.J. Worman (Columbia University), respectively.

### Cell cultures

Culture conditions and serum starvation/aphidicolin treatment were described before [7]. 2ME2 (1.3 µg/ml; Sigma M6383) or nocodazole (15 µg/ml; Sigma M1404) were added 16 hr prior to imaging. Reversine (5 µM; Sigma R3904) was added to 2ME2-containing media when indicated. RFP-lamin A and RFP-histone H2A fusions were used to generate cell lines with labeled NE and chromosomes, respectively (Supporting Information).

### Infections and live-cell imaging

Generation of, and infection with, the 1xMycR virus were described before [7]. To quantify infectivity of chimeric virions, 293T cells were co-transfected with plasmids expressing the pQCXIP-gfp-C1vector (2 µg), and the indicated molar ratios of wt MLV and MA-GFP/p12-CA-NC. A 1∶1 molar ratio represents 10 µg of pNCS and 5 µg of MA-GFP/p12-CA-NC plasmid. 48 hr post-transfection, supernatants of transfected cultures were filtered (0.45 µ), supplemented with HEPES (pH 7.0; 50 mM final concentration) and virus content was normalized by exogenous RT assay [56]. Supernatants with an equal RT activity were used to infect NIH3T3 cells for 2 hr in the presence of polybrene (hexadimethrine bromide; 8 µg/ml). Two days post-infections the cells were analyzed by FACS for the percentage of GFP^+^ cells. For live-cell imaging, chimeric particles were generated by co-transfecting 293T cells with a 1∶1 ratio of the indicated virus and the modified Gag as described above. Infections were carried out as described above with the following modifications: ∼8 hr before infection, the cells were plated (∼10% cofluency) in a 4-compartments-cell-view-glass-bottom-dish (35-mm; Greiner Bio One) and were infected with MOI of approximately 10 (based on the comparison of the RT activity of the samples to a standard MLV stock). For imaging, HEPES pH 7.0 (20 mM) was added to growth medium. Imaged samples were maintained at 37°C and supplied with CO_2_ when imaging exceeded 2 hr. In some experiments, chromosomes were stained with Hoechst 33342 (bisBenzimide H 33342 trihydrochloride, Sigma B2261; 1 µg/ml, 15 min, 37°C). Imaging was with spinning disk confocal (Yokogawa CSU-22 Confocal Head) microscope (Axiovert 200 M, Carl Zeiss MicroImaging), 100× lens (NA 1.45, Zeiss) and Evolve or HQ2 (Photometrics) cameras.

Single-cycle infection assays were carried out with the pQCXIP-GFP-C1 or pQCXIN (Clontech) vectors. For VLPs carrying the wt p12 sequence the following plasmids were co-transfected into 293T cells: vector plasmid (10 µg), VSV-G expression plasmid (2.5 µg), pGag-PolGpt helper plasmid [57] (5 µg), and the plasmid expressing the modified Gag MA-GFP/p12-CA-NC (2.5 µg). The PM14 mutation was inserted to the p12 sequences of pGag-PolGpt helper plasmid (generating pGag-PolGpt/PM14) and of MA-GFP/p12-CA-NC plasmid (generating pMA-GFP/p12-CA-NC/PM14). These two plasmids were used to generate PM14 VLPs as described above for VLPs with the wt p12 sequences. The LANA31 sequence was inserted into pMA-GFP/p12-CA-NC or pMA-GFP/p12-CA-NC/PM14, generating pMA-GFP/p12-CA-NC/LANA31and pMA-GFP/p12-CA-NC/PM14/LANA31, respectively. To generate VLPs with LANA31 module, pairs of pMA-GFP/p12-CA-NC/LANA31 and pGag-PolGpt, or pMA-GFP/p12-CA-NC/PM14/LANA31and pGag-PolGpt/PM14, were co-transfected with VSV-G and vector plasmids, using the same plasmid ratio indicated above. NIH3T3 cells were infected with the resulting VLPs, normalized by exogenous RT assay, and drug-resistant colonies were selected with either puromycin (4 µg/ml), or G418 (1 mg/ml).

### Western blot, immunofluorescence and fluorescence analyses

For Western blotting, virions were purified through sucrose cushions using ultracentrifugation [7] and detected with anti-GFP monoclonal antibody (Covance, MMS-118R). Immunofluorescence and calculations of the co-localization degree between CA and p12, or CA and GFP-p12, and between the chromosomes and each of these proteins were performed as in [7]. Quantification of the overlap between GFP fluorescence and chromosomes (RFP fluorescence) was performed as was described before for measurements of the overlap between p12 and chromatin signals [7]. Reconstitution of 3D images was performed with SlideBook software, employing MIP (Fig. 1D) or X-Ray (Fig. S2) functions.

### Calculations of PICs retention

For the calculation of the spatial retention of PICs over time, time-lapse sequences were processed [NoNeighbors deconvolution, Laplacian 2D filtering, SlideBook software (Intelligent Imaging Innovations)]. Objects were identified through intensity-based segmentation, with no further selection for specific PICs. For each movie, the second, third or fourth frame were superimposed on the first frame; the areas of overlapping PICs identified, and the signal intensity in overlapping areas was presented as the percentage of total intensity of objects in the first frame.

## Supporting Information

Figure S1 **Co-localization of CA and GFP-p12 in interphase and mitotic cells.** wt GFP-infected U/R cells were immunostained with anti-CA antibodies (red), 12 hpi. Chromosomes were stained with DAPI (blue). Interphase (A) and mitotic (B) cells are shown. Bars represent 10 µm.
(TIF)

Figure S2 **Close association between mitotic chromosomes and wt GFP, but not PM14 GFP, derived PICs.** U/R/RFP-H2A cells were infected with either wt GFP (A) or PM14 GFP (B), fixed and imaged. Serial optical sections of mitotic chromosomes were reconstituted into 3D images, with a 10 µm grid. Chromosomes are in red; free and chromosome-imbedded PICs are in dark and light green, respectively.
(TIF)

Movie S1 **PICs cytoplasmic movement in an interphase cell.** Time-lapse microscopy analysis of PICs in aphidicolin-treated, wt GFP-infected, U/R/RFP-laminA cell. Arrowheads mark PICs with directed cytoplasmic movement. Rectangle surrounds a group of extracellular virions attached to the glass. Cells were visualized 18 hpi. The decrease in the red and green signals, shown in this movie and in the following movies, is due to fluorescence bleaching over time (indicated in seconds). Frequency: 1 frame per second (fps).
(MOV)

Movie S2 **Cytoplasmic movement versus docking to mitotic chromosomes of MLV PICs.** 2ME2-treated, wt GFP-infected, mitotic (A) and interphase (B) U/R/RFP-laminA cells were visualized by time-lapse microscopy 12 hpi. The same procedure was applied to mitotic and interphase (C and D, respectively) unsynchronized U/R/RFP-H2A cells; unsynchronized NIH3T3/RFP-H2A cells (E); unsynchronized, D184A GFP-infected U/R/RFP-H2A cells (G); and nocodazole-treated, wt GFP-infected, mitotic U/R/RFP-H2A cells (F). Frequency: 1, 1.2, 0.5, 0.4, 1.3, 0.9 and 1.1 fps for A to G, respectively.
(MOV)

Movie S3 **Transition from cytoplasmic movement to docking to chromosomes of MLV PICs in cells entering mitosis.** wt GFP-infected, U/R/RFP-H2A (A) and U/R/RFP-laminA (B) cells, were visualized by time-lapse microscopy 5 hpi as they entered mitosis. Arrowheads mark PICs docked to the chromosomes. Empty triangles mark gaps in the disassembled NE. Frequency: 0.2 fps for A and B.
(MOV)

Movie S4 **Transition from docking to chromosomes to intra-nuclear movement of MLV PICs in cells exiting mitosis.** Mitotic, 2ME2-arrested, wt GFP-infected U/R/RFP-H2A cells were visualized by time-lapse microscopy before (A) and after (B) the addition of Reversine. The same procedure was applied for infected, unsynchronized U/R/RFP-H2A cells before (C) and after (D) natural exit from mitosis; and for unsynchronized U/R/RFP-laminA cells, before and after exit from mitosis (E and F, respectively). The U/R/RFP-H2A cells that exit mitosis and that were infected with D184A GFP (G) are the same cells shown during mitosis in part F of Movie S2. Frequency: 1.8, 1.8, 0.9, 0.9, 1.1, 1.1 and 1.1 fps for A to G, respectively.
(MOV)

Movie S5 **PICs with mutant p12 proteins fail to dock to mitotic chromosomes.** Interphase (A) and mitotic (B) cells of unsynchronized, PM14 GFP-infected U/R/RFP-H2A culture were visualized by time-lapse microscopy 12 hpi. The same procedure was applied to visualize mitotic cells of unsynchronized, PM14-infected NIH3T3/RFP-H2A culture (C); 2ME2-arrested U/R cells, co-infected with wt mCherry and PM14 GFP (D); and unsynchronized U/R/RFP-H2A cells infected with S(61, 65)A GFP (E) or with S(61, 65)A/M63I GFP (F). Broken lined circle marks chromosomal region with moving PM14 PICs with no apparent docking (B). Arrowheads point to representative wt mCherry PICs with stable chromosomal docking (D), or to an S(61, 65)A GFP PIC showing only transient, unstable association with the chromosomes (E). Frequency: 0.4, 0.4, 1.2, 0.8, 1.2 and 1.2 fps for A to F, respectively. Scale bar, 10 µm.
(MOV)

Movie S6 **Docking to the chromosomes of PICs with p12 proteins containing the LANA31 peptide.** Unsynchronized U/R/RFP-H2A cells were infected with PM14/LANA31 GFP (A) or wt/LANA31 GFP (B) and mitotic cells were visualized by time-lapse microscopy 12 hpi. Unsynchronized U/R cells were co-infected with wt mCherry together with wt/LANA31 GFP and cells that exit mitosis (C) were visualized as above. Frequency: 1.2, 1.2 and 0.8 fps for A to C, respectively. Scale bar, 10 µm.
(MOV)

Text S1 **Supplementary methods for plasmids construction and generation of cell lines with labeled NE and chromosomes.**
(DOC)
