# Supplementary figures and images for: p12 Tethers the Murine Leukemia Virus Pre-integration Complex to Mitotic Chromosomes
Source: PLoS Pathog. 2012 Dec 27;8(12):e1003103. doi: 10.1371/journal.ppat.1003103 (PMC3531515; doi:10.1371/journal.ppat.1003103)

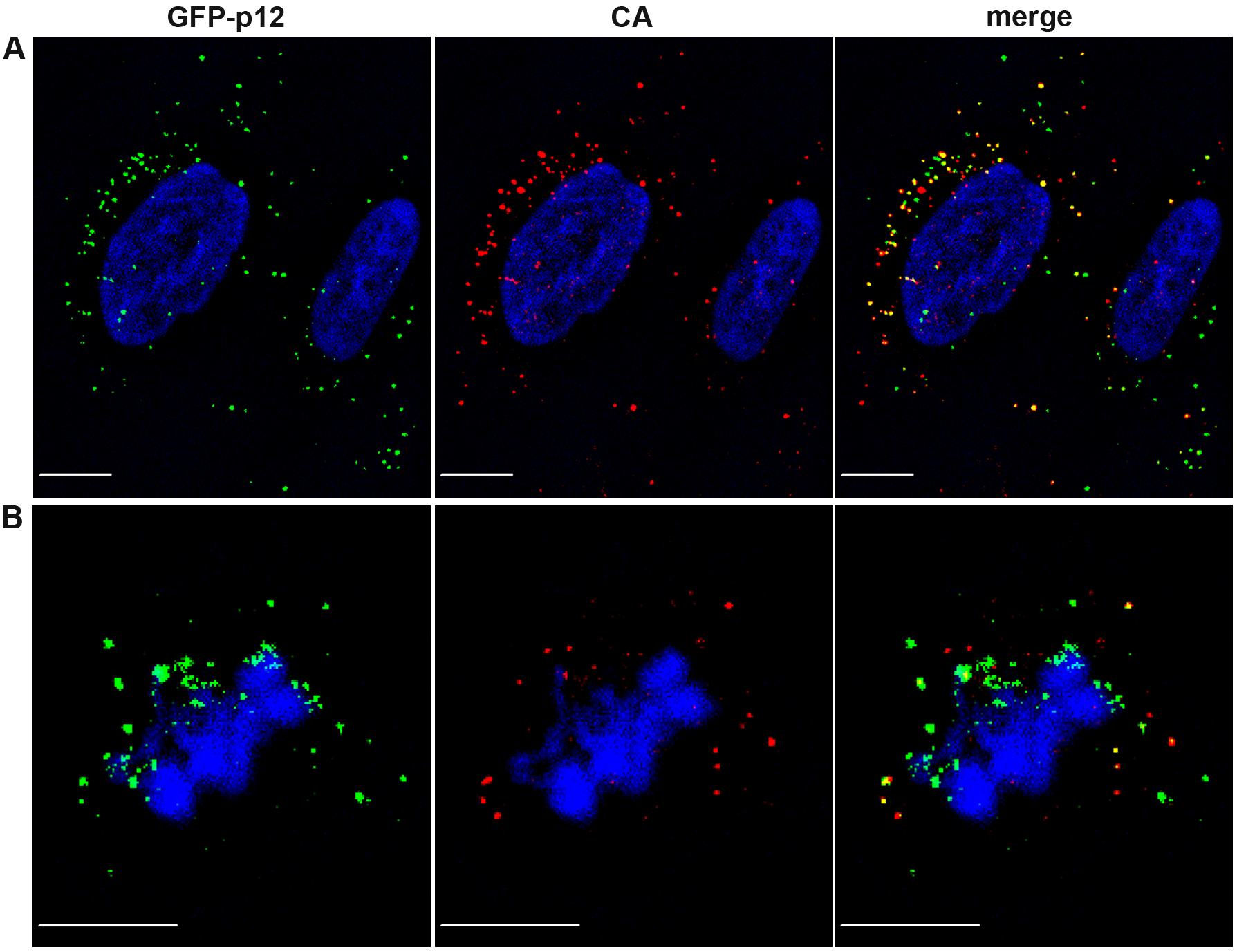

Supplement: Figure S1 — Co-localization of CA and GFP-p12 in interphase and mitotic cells. wt GFP-infected U/R cells were immunostained with anti-CA antibodies (red), 12 hpi. Chromosomes were stained with DAPI (blue). Interphase (A) and mitotic (B) cells are shown. Bars represent 10 µm. (TIF) [file ppat.1003103.s001.tif]

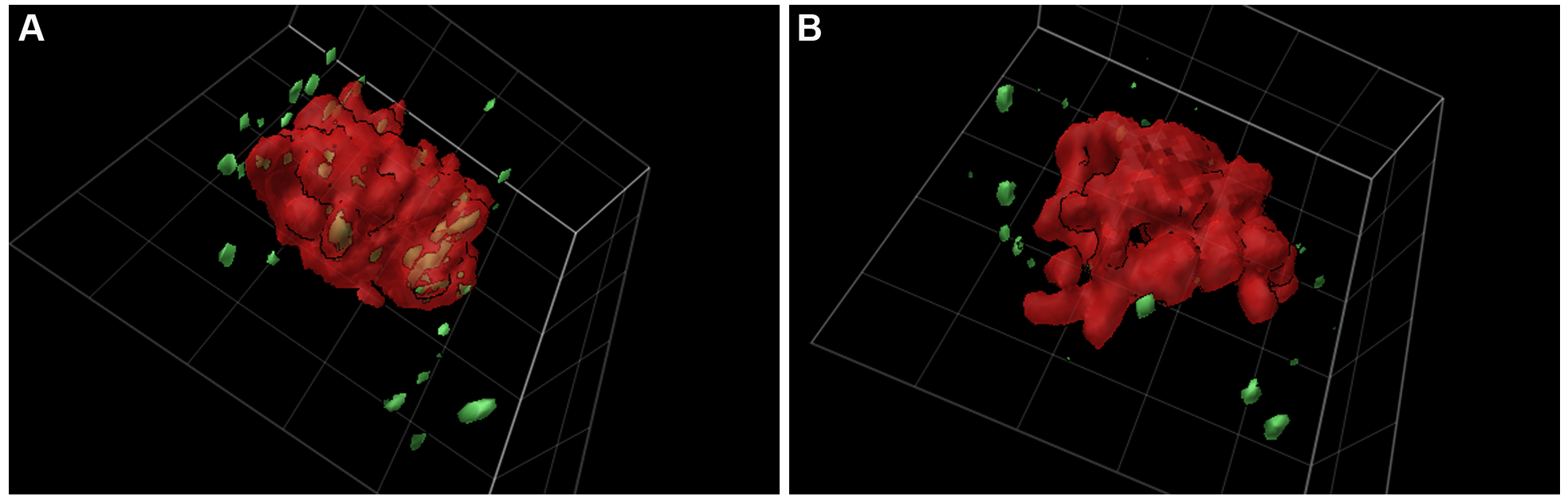

Supplement: Figure S2 — Close association between mitotic chromosomes and wt GFP, but not PM14 GFP, derived PICs. U/R/RFP-H2A cells were infected with either wt GFP (A) or PM14 GFP (B), fixed and imaged. Serial optical sections of mitotic chromosomes were reconstituted into 3D images, with a 10 µm grid. Chromosomes are in red; free and chromosome-imbedded PICs are in dark and light green, respectively. (TIF) [file ppat.1003103.s002.tif]
