# Supplementary material for: p12 Tethers the Murine Leukemia Virus Pre-integration Complex to Mitotic Chromosomes
Source: PLoS Pathog. 2012 Dec 27;8(12):e1003103. doi: 10.1371/journal.ppat.1003103 (PMC3531515; doi:10.1371/journal.ppat.1003103)
Supplement: Text S1 — Supplementary methods for plasmids construction and generation of cell lines with labeled NE and chromosomes. (DOC) [file ppat.1003103.s009.doc]

Plasmids construction

Overlapping PCR was used to generate the sequences of the modified Gag molecules MA-GFP/p12-CA-NC, MA-GFP-p12-CA-NC or MA-mCherry/p12-CA-NC, and to attach NotI and XbaI restriction sites to the 5’ and 3’ termini, respectively. These sequences were cloned into the same restriction sites of pCDNA3.1/Myc-His(+)A (Invitrogen) (note that the Myc and His epitopes of this plasmid are not expressed because of a stop codon present in the insert, upstream to the XbaI site). In each of these constructs, the sequence encoding the first six amino acids of p12 (PALTPS) was inserted, in-frame, between the C-terminus of MA and the N-terminus of GFP or mCherry, to generate a protease-cleavable MA-p12 site upstream of the fluorescent proteins. In the MA-GFP/p12-CA-NC or MA-mCherry/p12-CA-NC constructs, a sequence encoding for a protease-resistant linker (GGSI) was inserted, in-frame, between the C-terminus of GFP or mCherry, respectively, and the N-terminus of p12, to create protease-resistant fusions between p12 and the fluorescent proteins. In the control MA-GFP-p12-CA-NC construct, a sequence encoding for the terminal five residues of MA (RSSLY) was inserted, in-frame, between the C-terminus of GFP and the N-terminus of p12, to create a protease-cleavable MA-p12 junction downstream of GFP.

Generation of cell lines with labeled NE and chromosomes

U/R cells were transfected with pRFP-lamin A plasmid (expressing the RFP-lamin A fusion and the neo gene) and clones with NE marked by red fluorescence were isolated after G418 selection. Clone #1, named U/R/RFP-laminA, was used in the described experiments. U/R or NIH3T3 cells were co-transfected with pGK-puro plasmid (expressing the PAC gene) and pEF-H2AmRFP plasmid, and clones with chromosomes marked by red fluorescence were isolated after puromycin selection. Clones D6 (NIH3T3) and #2 (U/R) were named NIH3T3/RFP-H2A and U/R/RFP-H2A, respectively, and were used in the described experiments.
